# Temperature Dependence of the Number of Defect-Structures in Poly(vinylidene fluoride)

**DOI:** 10.3390/molecules29071551

**Published:** 2024-03-29

**Authors:** Jan Schwaderer, Marco Drache, Sabine Beuermann

**Affiliations:** Institute of Technical Chemistry, Clausthal University of Technology, Arnold-Sommerfeld-Straße 4, 38678 Clausthal-Zellerfeld, Germany; jan.schwaderer@tu-clausthal.de (J.S.); marco.drache@tu-clausthal.de (M.D.)

**Keywords:** PVDF, defect structure, temperature dependence

## Abstract

Poly(vinylidene fluoride) (PVDF) is predominantly characterized by alternating CH_2_ and CF_2_ units in a polymer backbone, originating from the head-to-tail addition of monomers or regular propagation. Due, to a small extent, to inverse monomer addition, so-called defect structures occur which influence the macroscopic properties of PVDF significantly. The amount of defect structures in the material is determined by the polymerization conditions. Here, the temperature dependence of the fraction of defect structures in PVDF obtained from polymerizations between 45 and 90 °C is reported. We utilized ^19^F-NMR spectroscopy to determine the fraction of defect structures as a function of temperature. To derive kinetic data, the polymerization of VDF is considered a quasi-copolymerization described by the Terminal Model involving four different propagation reactions. Based on the experimentally determined temperature-dependent fractions of defect structures, the known overall propagation rate coefficient, and taking into account the self-healing behavior of the macroradical, the Arrhenius parameters of the individual propagation rate coefficients were determined using the Monte Carlo methods.

## 1. Introduction

Poly(vinylidene fluoride) (PVDF) is a thermoplastic fluoropolymer. It is characterized by high thermal stability and resistance to acids and UV radiation, as well as low flammability [1,2] and a degree of crystallinity between 35 and 70% [3]. Due to its exceptional properties, PVDF is used in a wide range of applications, including valves, pumps, paints, and insulation materials [1]. In addition, the crystalline fraction of PVDF can exist in five different polymorphic phases [4,5]. The β phase is particularly interesting, because it possesses a macroscopic dipole moment, thereby exhibiting piezoelectric, ferroelectric, and pyroelectric properties [5,6]. Thus, PVDF with a high β-phase content is of interest for electronic components, solar panels, and sensors [7,8]. Because of its unique spectrum of properties, PVDF is the second most used fluoropolymer [9].

**Scheme 1:**
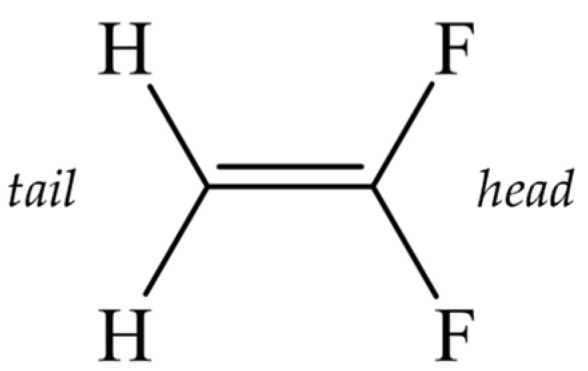
Chemical structure of VDF.

The microstructure of PVDF is described by its molar mass distribution, the degree of branching, and the proportion of defect structures. These structural characteristics are strongly influenced by the choice of process conditions for radical polymerization. For example, by employing controlled radical polymerization techniques, PVDF with narrow molar mass distributions (MMDs) and a small number of branch points is obtained [10,11], while free radical polymerizations lead to broader MMDs and a higher degree of branching. The polymer microstructure significantly impacts its properties, including the degree of crystallization [12], miscibility [13], and the type of polymorph formed [14,15]. This work deals with the influence of temperature on the fraction of defect structures. This structural motif originates from the fact that VDF is an asymmetric monomer that consists of a head (CF_2_ group,) and a tail (CH_2_ group), as shown in Scheme 1.

**Scheme 2:**
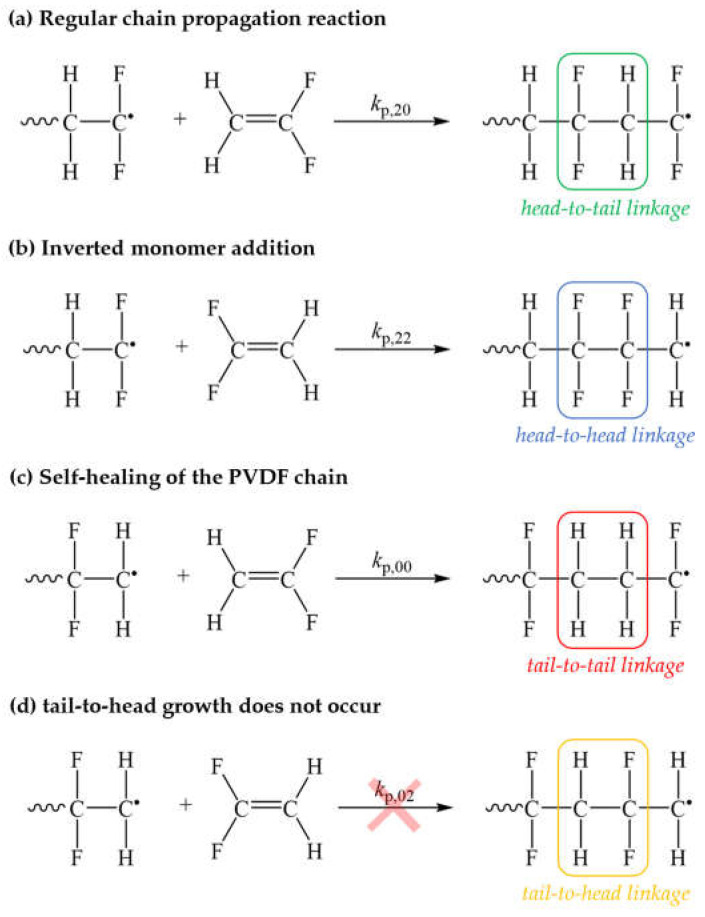
The quasi-copolymerization of VDF is described by the regular and inverse incorporation of monomers via four distinct propagation reactions according to the terminal model.

Upon addition of a radical to the monomer, the radical functionality is preferably located at the CF_2_ group. If the addition of the next VDF unit occurs in a manner that preserves the CF_2_ radical functionality at the chain end, a head-to-tail linkage is formed. This propagation step is referred to as regular propagation of the PVDF macroradical and occurs preferentially during polymerization. This reaction leads to a polymer backbone consisting of alternating CF_2_ and CH_2_ groups, as illustrated in the upper part of Scheme 2. However, other propagation reactions are feasible, too. A change in radical functionality occurs if a macroradical with a terminal CF_2_ group adds to the head side of the VDF molecule and a head-to-head linkage is created. This irregular addition of monomer leads to so-called defect structures −CF_2_−CF_2_− in the polymer backbone (second reaction in Scheme 2). According to IUPAC recommendations, the terms regioregular and regioirregular are used for head-to-tail and head-to-head linkages, respectively [16]. Since the majority of all previous publications related to PVDF use the term “defect structure”, this term is used in the following. Defect contents between 5% and 10% were reported [3,16,17,18,19,20,21]. The fraction of defect structures influences the degree of crystallinity, toughness, mechanical strength, and impact resistance of PVDF [7]. In addition, to obtain high fractions of β-phase material the number of defect structures should be low [5,22].

Irregular propagation results in a terminal CH_2_ radical functionality, which in turn enables two additional growth reactions. In the first case, the preferred CF_2_ radical functionality is restored, resulting in a tail-to-tail linkage, as illustrated in part c of Scheme 2. This growth reaction is called defect healing and, in the case of free radical VDF polymerization, always follows irregular propagation [23]. This phenomenon is also referred to as self-healing of the PVDF chain. As a result, a tail-to-head linkage cannot be formed (see lower part of Scheme 2).

The following terminology is introduced for naming the carbon units. Each carbon atom is coded by a digit, where the digit value corresponds to the number of fluorine atoms directly attached to that carbon atom. Carbon atoms that do not carry fluorine atoms are abbreviated with the digit 0 (CH_2_). Therefore, the number 2 represents a carbon atom with two directly bonded fluorine atoms (CF_2_). These naming conventions result in the propagation coefficients *k*_p,20_ for regular growth (head-to-tail growth), *k*_p,22_ for defect growth (head-to-head-growth), *k*_p,00_ for defect healing (tail-to-tail growth), and *k*_p,02_ for tail-to-head growth, which, in this case, assumes a value of 0.

**Scheme 3:**
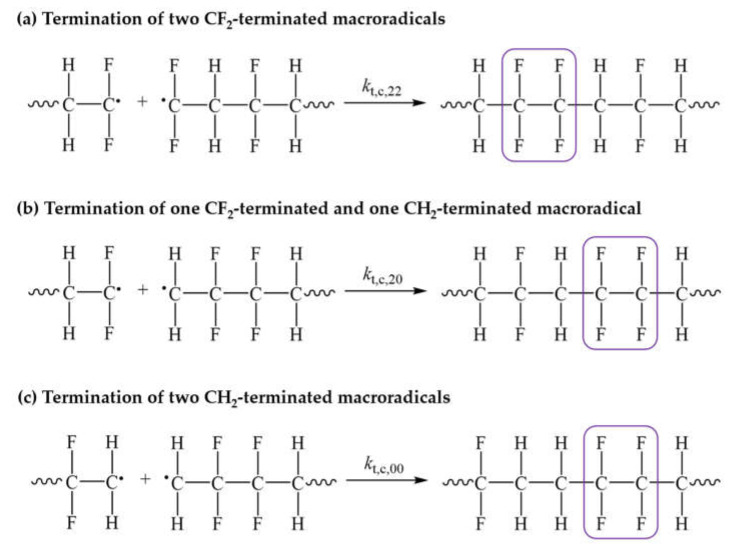
The defect structure −CF_2_−CF_2_− can also result from termination by combination.

In the case of VDF polymerizations, termination occurs exclusively through combination [19,24], which suggests that, in principle, a relatively large fraction of the defect structure could arise from bimolecular termination, as illustrated in Scheme 3. The structural motif −CH_2_−CF_2_−CH_2_−CF_2_−CF_2_−CH_2_−CF_2_−CH_2_−CF_2_− formed via termination reactions (a) and (b) in Scheme 3, was not detected in the ^19^F-NMR spectrum. The termination of two macroradicals with terminal CH_2_ radical functionalities (termination reaction (c)) is unlikely since only a very small number of these species is present in the reaction mixture. For these reasons, in general, it is assumed that the contribution of termination to the fraction of CF_2_−CF_2_ structures is negligible.

In the context of this work, PVDF which was obtained by a rather special type of polymerization known as pulsed laser-initiated polymerization (PLP) was analyzed [25,26]. Generally, PLP is characterized by the generation of initiator-derived radicals by applying an evenly-spaced sequence of short UV laser pulses to a mixture consisting of monomer and photoinitiator [27]. Additionally, a solvent undergoing little chain transfer may be present. Since a high concentration of initiator radicals, typically in the order of 10^−6^ L∙mol^−1^∙s^−1^, is generated at a certain moment in time, the probability of termination events at this moment in time is significantly enhanced. Consequently, the termination of an initiator-derived radical and a macroradical is favored. The likelihood of a bimolecular chain termination reaction, where two growing chains combine, is significantly reduced [28]. Moreover, the probability of chain propagation is significantly higher than of chain termination due to the significantly higher monomer concentration compared to the radical concentration. Thus, as discussed for the general case of VDF radical polymerizations, for polymers obtained from PLP, termination as a source of CF_2_−CF_2_ defects is also negligible.

## 2. Results and Discussion

**Figure 1:**
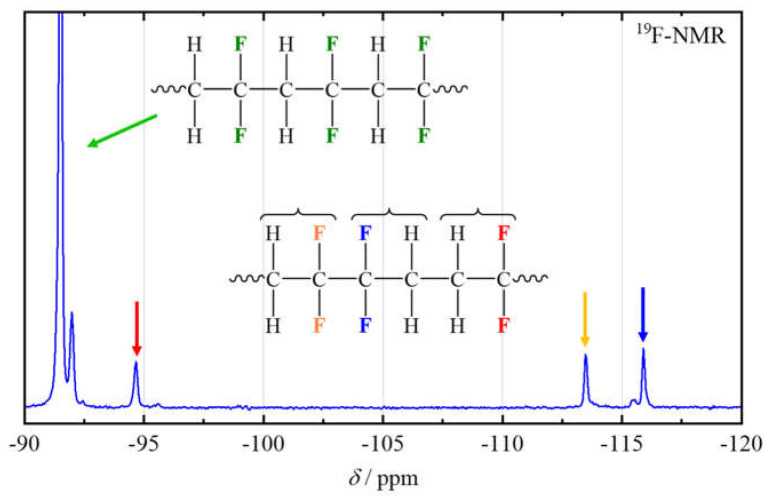
^19^F-NMR spectrum of a typical PVDF sample.

The fraction of defect structures in PVDF is calculated using ^19^F-NMR spectroscopy. At a chemical shift of −91.4 ppm, the signal of regular PVDF growth is detected [29]. Three additional signals are observed, at −94.5 ppm, −113.8 ppm, and −116.2 ppm. These signals are assigned to the fluorine atoms of CF_2_ groups located in the immediate vicinity of defect propagation [29]. In Figure 1, an exemplary ^19^F-NMR spectrum and the associated structural motifs are shown. The orange marked signal at −113.8 ppm corresponds to the fluorine atoms of a CF_2_ group to which the following VDF unit is inversely added. The blue signal at −116.2 ppm is assigned to the fluorine atoms of an inversely incorporated VDF unit. At a chemical shift of −94.5 ppm, the red-marked signal corresponds to a VDF unit directly following a defect growth. This VDF unit, obtained through a tail-to-tail linkage, is attributed to defect healing of the PVDF chain.

To determine the self-healing degree of PVDF, the integrals of the signals of the defect-causing VDF units and the defect-healing VDF units can be put into proportion, as shown in Equation (1). *A_i_* denotes the integral of the respective signal at the chemical shift *δ* = *i* ppm in the ^19^F-NMR spectrum.

(1) $$\mathrm{degree}\;\mathrm{of}\;\mathrm{self}-\mathrm{healing}=\frac{A_{-116.2}}{A_{-94.5}}$$

**Figure 2:**
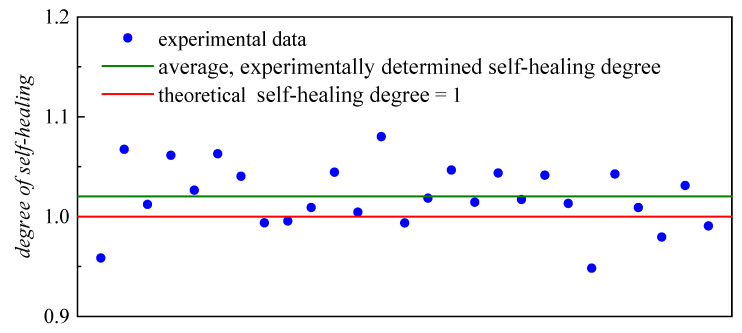
The degree of self-healing as determined experimentally and the average value of all individual data. The red line indicates the theoretical value of 1.

In Figure 2, it is shown that the experimentally derived data for the degree of self-healing are predominantly between 1.0 and 1.1, with an average value of 1.02. The *degree of self-healing* does not show a systematic variation with polymerization conditions (temperature, pressure) and molecular weight. Since the ^19^F-NMR spectra do not show any peaks that may be assigned to the above-mentioned termination products, the defect structures are due to inverse monomer incorporation. The content of defect structures in PVDF is calculated using Equation (2).

(2) $${\mathrm{defect}\;\mathrm{content}}_{\exp}=\frac{A_{-116.2}}{A_{-91.4}+A_{-94.5}+A_{-113.8}+A_{-116.2}}\;\text{×}100\%$$

Upon valuation of all the NMR data, a temperature dependence on the number of defect structures in the PVDF became evident. With the increasing polymerization temperature, a higher fraction of defect structures were generated in the PVDF, which is in line with previous reports [22]. In order to tailor PVDF with respect to the number of defect structures via kinetic modeling, knowledge of the temperature dependence of all kinetic coefficients for the reactions shown in Scheme 2 is required. While these individual coefficients are not accessible from experiments, the overall polymerization propagation rate coefficient *k*_p,app_ was already reported [25]. The values of *k*_p,app_ are related to the individual coefficients *k*_p,20_, *k*_p,22_, *k*_p,00_, and *k*_p,02_ using Equation (3), which was derived for the quantification of defect structures in poly(vinyl acetate) [30].

(3) $$k_{p,\mathrm{app}}=k_{p,20}-\frac{k_{p,20}-k_{p,02}-2\cdot k_{p,00}}{1+\frac{k_{p,00}}{k_{p,22}}}$$

As already discussed, based on Scheme 2, tail-to-head growth does not occur, thus the coefficient *k*_p,02_ is equal to 0, and Equation (3) simplifies accordingly. Furthermore, Siegmann et al. described the overall propagation rate coefficient of VDF for polymerizations in solution with supercritical carbon dioxide by Equation (4) over a wide range of temperatures and pressures [25].

(4) $$\ln k_{p,\mathrm{app}}=\ln A\;-\frac{E_{a}}{R\;\cdot \;T}\;-\frac{\Delta V^{‡}\cdot p}{R\cdot T}$$

with *p* introduced in bar and *T* in K. *A* represents the pre-exponential factor, *E*_a_ denotes the activation energy, *T* signifies the temperature, *p* stands for pressure, and *R* represents the universal gas constant. For VDF, the activation energy is found to be 30.2 kJ∙mol^−1^, the activation volume is −22.7 cm^3^∙mol^−1^, and the pre-exponential factor is 4.66·10⁸ L∙mol^−1^∙s^−1^ [25].

**Scheme 4:**
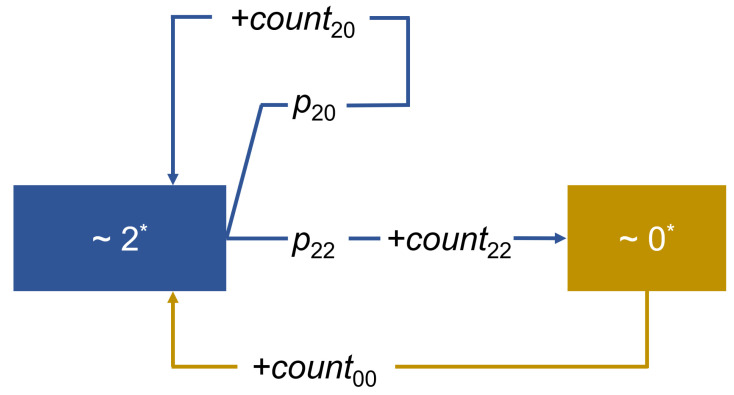
Scheme of polymer chain propagation simulation.

For calculating the defect fraction using the individual propagation rate coefficients *k*_p,00_, *k*_p,20_, and *k*_p,22_, a simple Monte Carlo simulation of chain growth was implemented in the Python programming language. An illustrated representation of the simulation is shown in Scheme 4. The corresponding Python script for the Monte Carlo simulations is provided in the Supporting Information as file pvdf-defect.txt. The addition of 10^7^ monomer units was recorded in the simulation. As a result, the program outputs the temperature-dependent values of *k*_p,app_ and the fraction of defects.

The simulation starts with a growing chain end “~2*”. The probability of regular growth *p*_20_ can be determined using the coefficients *k*_p,20_ and *k*_p,22_ according to Equation (5).

(5) $$p_{20}=\frac{{(k}_{p,20}/k_{p,22})}{{(k}_{p,20}/k_{p,22})+1}$$

Using a random number *r* (0 ≤ *r* ≤ 1), it is decided whether regular or irregular growth occurs at the chain end. If the random number is less than or equal to *p*_20_, regular growth occurs, and the counter *count*_20_ is incremented by 1. After this growth step, the chain end remains unchanged as “~2*”. The probability *p*_22_ is 1 − *p*_20_. Therefore, if the random number r is *r* > *p*_20_, the pathway of irregular growth is selected. The chain end changes to “~0*”, and the defect structure counter *count*_22_ increases by 1. Since instantaneous self-healing occurs, the chain end switches back to “~2*” in the subsequent propagation step, and the counter *count*_00_ is incremented by 1. For the simulation, 10^6^ addition steps were performed, and the defect fraction was calculated from the counters according to Equation (6).

(6) $${\mathrm{defect}\;\mathrm{fraction}}_{\mathrm{sim}}=\frac{{\mathrm{count}}_{22}}{{\mathrm{count}}_{22}+{\mathrm{count}}_{00}+{\mathrm{count}}_{20}}\text{×}100\%$$

The goal of the optimization is to achieve a good match of the defect fraction with experimental data. Therefore, the sum of squared differences is minimized while varying the coefficients *k*_p,00_, *k*_p,20_, and *k*_p,22_. A constraint is that the *k*_p,app_ calculated using the determined individual coefficients matches the experimental data within the experimental error limits. For this purpose, a stochastic Metropolis–Hastings algorithm was used, as described in detail by Feuerpfeil et al. [31]. The result of the optimization is presented in Table 1. The activation energy of the regular monomer addition, *E*_a,20_ = 25.5 kJ∙mol^−1^, is significantly lower than for the inverse propagation, *E*_a,22_ = 30.7 kJ∙mol^−1^, which explains the increase in defect structures at higher temperatures. The temperature dependence of the self-healing reaction is the most pronounced, as indicated by the activation energy, *E*_a,00_, of 35.4 kJ∙mol^−1^. The pre-exponential factor of the inverse propagation reaction is the lowest, which is in line with the small fraction of defect structures. For example, the kinetic coefficients *k*_p,20_, *k*_p,22_, and *k*_p,00_ calculated for 45 °C with the Arrhenius parameters according to *k* = *A*∙exp(−*E*_a_/R*T*) are 12,900 L∙mol^−1^∙s^−1^, 592 L∙mol^−1^∙s^−1^, and 5430 L∙mol^−1^∙s^−1^, respectively. As expected, the predominantly occurring regular head-to-tail addition is the fastest reaction, and the associated *k*_p,20_ is higher by a factor of 22 than the kinetic coefficient *k*_p,22_ for defect growth. The self-healing reaction coefficient is a factor of two lower than for the regular chain propagation. At 90 °C the differences between *k*_p,20_ and *k*_p,22_ are slightly lower due to the higher activation energy of *k*_p,22_, with *k*_p,20_ being 17 times higher than *k*_p,22_.

**Figure 3:**
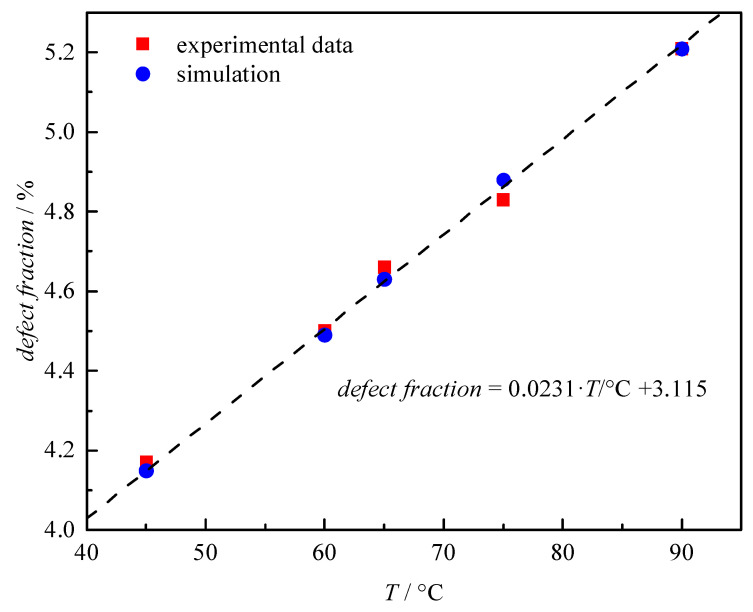
Experimental and simulated defect fraction of PVDF as a function of temperature.

In Table 2 and Figure 3, a very good agreement between the calculated fraction of defect structures and the experimental data are shown. Table 2 also demonstrates that the constraint regarding *k*_p,app_ is met. A linear dependence of the fraction of defect structures with increasing reaction temperature is observed, as shown in Figure 3. The fraction of defect structures determined from the NMR spectra is in good agreement with the literature reports. For example, Guerre et al. reported a percentage of 4.1% for the defect structures of PVDF from RAFT (reversible addition fragmentation transfer) polymerizations at 75 °C, resulting in polymers with rather low average molar masses, *M*_n_, of, at most, 13,800 g∙mol^−1^ [16]. It was pointed out that the fraction of defects increases with conversion and polymer molar mass. In contrast to the literature data, the results in Table 2 were obtained for polymers from free-radical polymerizations with significantly higher molar masses, e.g., with *M*_n_ being higher than 40,000 g∙mol^−1^. Therefore, the slightly higher defect fractions in Table 2 are due to the higher PVDF molar mass.

Moreover, it is important to note that the largely varying activation energies of the various propagation reactions are important for the data evaluation. The VDF system is not suited for a description of the defect structures based on ratios of propagation rate coefficients, e.g., comparable to the previously reported use of reactivity ratios in the case of vinyl acetate polymerizations [32].

## 3. Materials and Methods

VDF is polymerized via pulsed laser-induced polymerization (PLP) in homogeneous phase with supercritical CO_2_ as the reaction medium in an optical high-pressure cell [33]. The reaction mixture consisting of VDF (99%, provided by 3M/Dyneon, Burgkirchen an der Alz, Germany) and carbon dioxide (CO_2_, ≥99% by volume, Westfalen AG, Münster, Germany), and the photoinitiator 2,2-dimethoxy-2-phenylacetophenone (DMPA, 99%, Acros Organics, Geel, Belgium) is prepared and transferred into the optical high-pressure cell as reported previously [25]. PLP experiments are carried out at temperatures of 45 °C, 60 °C, 65 °C, 75 °C, and 90 °C at a pressure of 1050 bar. An excimer laser (ExiStar XS 500, Coherent, Santa Clara, CA, USA) operated at a wavelength of 351 nm with a pulse repetition rate adjustable between 1 and 500 Hz is used. Details of the pulsed-laser-initiated polymerizations in solution with supercritical CO_2_ and the measurements of the molar mass distributions using size-exclusion chromatography are provided elsewhere [25,26]. As solvents for the NMR measurements with a Bruker AVANCE 400 MHz spectrometer, deuterated N,N-dimethylformamide (DMF-d_7_, 99.5%, Deutero GmbH, Kastellaun, Germany) and acetone-d_6_ (99.8%, Deutero GmbH) are used.

It is important to note that, in the case of the PLP of VDF in solution with supercritical CO_2_, only little variation in temperature below 1 °C may be achieved due to the design of the reaction cell [28]. Moreover, applying the laser pulses in several sequences, the presence of supercritical CO_2_ as a solvent, and stopping the reaction at a monomer conversion below 5%, all contribute to good temperature control, too. Further, the presence of CO_2_ does not affect the propagation rate coefficients [34,35].

## 4. Conclusions

The polymerization temperature has a significant impact on the proportion of defect structures in PVDF, and thus, on the thermal and mechanical properties of the polymer. For this reason, it is important to precisely control the reaction temperature during synthesis. Based on ^19^F-NMR spectroscopy, the fraction of defect structures was calculated, which is well-expressed by a linear relation. In addition, a Monte Carlo simulation was performed. The fraction of defect structures from the simulation matches very well with the experimental data, and the constraint, which is the good agreement of *k*_p,app,sim_ and *k*_p,app,exp_, is well maintained. The individual propagation rate coefficients *k*_p,20_, *k*_p,22_, and *k*_p,00_ are determined as a function of temperature, with the associated activation energies being largely different. Particularly important is the activation energy of 30.7 kJ∙mol^−1^ for the inverse propagation reaction, whereas a significantly lower value of 25.5 kJ∙mol^−1^ is derived for the regular propagation reaction. The individual rate coefficients with temperature dependence are required to assist in tailoring the fraction of defect structures in the product using simulations.

## Figures and Tables

**Table 1:** Determined activation energies and pre-exponential factors.

*A*_20_	*A*_22_	*A*_00_	*E*_a,20_	*E*_a,22_	*E*_a,00_
L∙mol^−1^∙s^−1^				kJ∙mol^−1^	
1.99 × 10^8^	6.50 × 10^7^	3.52 × 10^9^	25.5	30.7	35.4

**Table 2:** Experimentally determined defect contents and calculated average growth rate coefficients *k*_p,app_ at different polymerization temperatures.

*T*/°C	*defect fraction*_exp_/%	*defect fraction*_sim_/%	*k*_p,app,exp_ /L·mol^−1^·s^−1^	*k*_p,app,sim_ /L·mol^−1^·s^−1^
45	4.2	4.2	11,200	12,700
60	4.5	4.5	20,000	19,900
65	4.7	4.6	23,300	23,000
75	4.8	4.8	30,900	30,100
90	5.2	5.2	46,000	43,800

## Data Availability

Data are contained within the article.

## Supplementary Materials

The following supporting information can be downloaded at: https://www.mdpi.com/article/10.3390/molecules29071551/s1, pvdf-defect.txt.
